# Machine Learning for Onset Prediction of Patients with Intracerebral Hemorrhage

**DOI:** 10.3390/jcm12072631

**Published:** 2023-03-31

**Authors:** Thilo Rusche, Jakob Wasserthal, Hanns-Christian Breit, Urs Fischer, Raphael Guzman, Jens Fiehler, Marios-Nikos Psychogios, Peter B. Sporns

**Affiliations:** 1 Department of Neuroradiology, Clinic of Radiology & Nuclear Medicine, University Hospital Basel, 4031 Basel, Switzerland; 2 Department of Neurology, University Hospital Basel, 4031 Basel, Switzerland; 3 Department of Neurosurgery, University Hospital Basel, 4031 Basel, Switzerland; 4 Department of Diagnostic and Interventional Neuroradiology, University Medical Center Hamburg-Eppendorf, 55131 Hamburg, Germany; 5 Department of Radiology and Neuroradiology, Stadtspital Zürich, 8063 Zürich, Switzerland

**Keywords:** artificial intelligence, onset prediction, intracerebral hemorrhage, machine learning

## Abstract

Objective: Intracerebral hemorrhage (ICH) has a high mortality and long-term morbidity and thus has a significant overall health–economic impact. Outcomes are especially poor if the exact onset is unknown, but reliable imaging-based methods for onset estimation have not been established. We hypothesized that onset prediction of patients with ICH using artificial intelligence (AI) may be more accurate than human readers. Material and Methods: A total of 7421 computed tomography (CT) datasets between January 2007–July 2021 from the University Hospital Basel with confirmed ICH were extracted and an ICH-segmentation algorithm as well as two classifiers (one with radiomics, one with convolutional neural networks) for onset estimation were trained. The classifiers were trained based on the gold standard of 644 datasets with a known onset of >1 and <48 h. The results of the classifiers were compared to the ratings of two radiologists. Results: Both the AI-based classifiers and the radiologists had poor discrimination of the known onsets, with a mean absolute error (MAE) of 9.77 h (95% CI (confidence interval) = 8.52–11.03) for the convolutional neural network (CNN), 9.96 h (8.68–11.32) for the radiomics model, 13.38 h (11.21–15.74) for rater 1 and 11.21 h (9.61–12.90) for rater 2, respectively. The results of the CNN and radiomics model were both not significantly different to the mean of the known onsets (*p* = 0.705 and *p* = 0.423). Conclusions: In our study, the discriminatory power of AI-based classifiers and human readers for onset estimation of patients with ICH was poor. This indicates that accurate AI-based onset estimation of patients with ICH based only on CT-data may be unlikely to change clinical decision making in the near future. Perhaps multimodal AI-based approaches could improve ICH onset prediction and should be considered in future studies.

## 1. Introduction

Intracerebral hemorrhage (ICH) has an incidence of 10–30 per 100.000 people per year worldwide [1,2]. The by far most common form is spontaneous primary ICH, caused by rupture of small intraparenchymal arterial vessels and arterioles caused by preexisting vascular damage in the setting of chronic arterial hypertension or amyloid angiopathy [3]. Compared with ischemic strokes, hemorrhagic strokes have a higher morbidity, and an overall mortality of more than 30% in the first 30 days [3,4,5]. Moreover, half of ICH-associated mortality occurs during the first 24 h after the initial event [6], and a poor outcome with persistent physical impairment in 75% of all patients after 1 year [7]. Therefore, ICH results in a significant financial burden on the health care system due to the long-term treatment that is often required [8,9,10].

In many patients diagnosed with ICH by imaging, the exact onset is unknown [11]. However, therapeutic and triage decisions are based on factors such as the likelihood of rebleeding, brain edema, and herniation which are associated with the time since onset of the bleeding [12]. Thus, it has been shown that functional outcomes after 30 days are significantly worse in patients with unclear onset compared to patients with a known onset [13]. Possible treatment strategies [14] such as blood pressure reduction [15,16,17], neurosurgical evacuation [18], and application of tranexamic acid [19] may reduce the risk of hematoma expansion associated with neurological deterioration leading to poor outcomes and death [12,20]. Even though promising manual [12,20,21,22] and machine learning based algorithms have been developed and tested to predict the risk of hematoma growth and functional outcomes [23], to our knowledge there is no study that tested the reliability of machine-learning algorithms for onset estimation of patients with ICH based on imaging features. An AI-based approach is therefore particularly interesting and promising, as it can detect image information that may be hidden or primarily not obvious and use it for analysis processes. This opens new possibilities for data analysis. In recent years, AI-based applications have increasingly found their way into clinical routine, especially in the field of radiology [24,25]. For example, the automated detection of pulmonary artery emboli in CT scans of the thorax [26] or the detection and volumetry of ICH in CT scans of the head [27,28,29].

Therefore, we hypothesized that onset estimation in patients with ICH using machine learning may be feasible and may be more accurate than human readers. 

## 2. Methods

### 2.1. Study Design

This study includes all consecutive patients diagnosed with ICH by computed tomography (CT) at the University Hospital Basel between January 2007–July 2021. Patients were identified using a self-programmed tool for internal Picture Archiving and Communication System (PACS)-based data retrieval (PACS crawler). Further inclusion criteria were being an age > 17 years at the timepoint of imaging and having a known onset of symptoms. Patients who had declined the use of personal data for research purposes were excluded.

### 2.2. Data Processing, Classifier Training and Image Assessment

CT datasets were exported to a research server (NORA Imaging Platform, Freiburg, Germany [10.1055/s-0037-1602977]). Manual segmentation of the hematoma in 1 mm thick axial slices (soft tissue window) was performed in a random subset of 319 CT datasets to train an algorithm for automatic ICH segmentation. All other CT datasets were segmented using the trained classifier (as described below).

Clinical data including time points of symptom onset were extracted from patients’ clinical records. 

For the automated segmentation of ICHs, we trained a nnU-Net on our manually annotated dataset [https://arxiv.org/abs/1809.10486, accessed on 10 September 2021] [10.1038/s41592-020-01008-z]. nnU-Net is a medical segmentation framework, which automatically configures the data preprocessing as well as the hyperparameters for training a U-Net. It can derive heuristics for optimally setting the data preprocessing parameters (e.g., normalization and resampling) as well as the U-Net configuration (e.g., number of layers and batch size) based on the characteristics of the input dataset. Furthermore, it performs extensive data augmentations (image rotation, blurring, etc.). On more than 20 public imaging segmentation challenges, this automatically configured segmentation pipeline was superior to other submissions. For this reason, the nnU-Net was used for the ICH segmentation.

**Figure 1:**
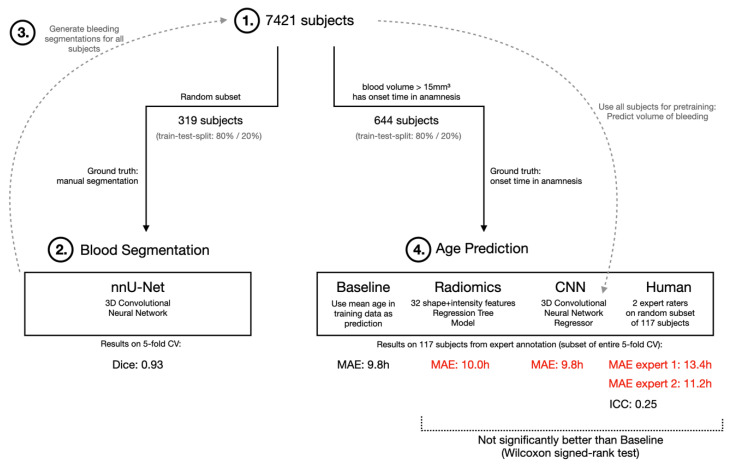
Flowchart of inclusion of patients. Overview study design: On a subset of the original study population (1.) a hematoma segmentation model is trained (2.) which is used to generate hematoma segmentations for the entire study population (3.) Then, the radiomics-, CNN-, and human-based age prediction is performed (4.) (MAE: Mean absolute error; CNN: Convolutional Neural Network, nnU-Net: Neural network for semantic segmentation).

For estimation of the onset of the patients with ICH, two different algorithms were tested: 1. Regression trees with radiomics features (Radiomics). 2. Convolutional neural network (CNN). The classifiers were trained based on the gold standard of 644 datasets with (a) a known onset of >1 h and <48 h and (b) a hematoma volume of >15 mm^3^ (see flowchart in Figure 1). Criterion (a) was defined to include only acute to subacute ICHs in the training dataset as therapeutic and triage decisions are particularly relevant in this time frame. Criterion (b) was defined because a cut-off of >15 mm^3^ provided reliable automated ICH-segmentation. A 5-fold cross-validation was used for evaluation. No extra validation set for hyperparameter optimization was used since only standard models and hyperparameters were used.

The radiomics approach consisted of 32 radiomics features (14 shape features: elongation, flatness, least axis length, major axis length, max 2d diameter column, max 2d diameter row, max 2d diameter slice, max 3d diameter, mesh volume, minor axis length, sphericity, surface area, surface volume ratio, voxel volume; 18 first order intensity features: 10th percentile, 90th percentile, energy, entropy, interquartile range, kurtosis, maximum, mean absolute deviation, mean, median, minimum, range, robust mean absolute deviation, root mean squared, skewness, total energy, uniformity, variance), which were extracted from the CT images and the ICH segmentation using pyradiomics [10.1158/0008-5472.CAN-17-0339]. Those features were used to train a gradient boosted regression tree using XGBoost [https://doi.org/10.1145/2939672.2939785, accessed on 10 September 2021] (learning rate 0.01, number of trees 200, maximum depth 2. For the CNN approach, a 3d convolutional neural network regressor was trained. As architecture the CBR-tiny model from [https://papers.nips.cc/paper/2019/hash/eb1e78328c46506b46a4ac4a1e378b91-Abstract.html, accessed on 10 September 2021] was used by replacing the 2D convolutions by 3D convolutions (network architecture: 4 sequential blocks of Convolution + BatchNorm + MaxPooling followed by adaptive average pooling and a fully connected layer). To improve the model performance, it was pretrained on a large dataset with an auxiliary task: on 7421 CT images containing ICHs the network was trained to predict the volume of the ICH (Figure 1). The volume was derived from the ICH segmentation generated by our segmentation model. After this first training, the model was finetuned on the estimation of onset task. For training, the following hyperparameters were used: batch size 8, learning rate 0.0005, number of epochs 15, learning rate decay to 0 over the entire training with cosine scheduler.

For both the segmentation as well as the onset prediction, a 5-fold cross-validation was used for evaluation.

In addition, a random subset of 117 CT datasets (Figure 1) was selected from the training dataset and analyzed by two radiologists (4 and 5 years of experience in ICH interpretation) regarding hemorrhage age based on their clinical–radiological experience and secondary factors (perifocal edema: relatively large edema = advanced bleeding age; Hounsfield Units (HU): acute bleeding approximately 60–70 HU, approximately 2 HU drop per 24 h). The hemorrhage age was given in hours. An interrater comparison and a comparison with the results of the AI-based age prediction were performed. 

### 2.3. Statistics

For the calculation of confidence intervals, we used bootstrapping with 10,000 random permutations and for the comparison of human raters as well as of the AI algorithms with the baseline we used Wilcoxon signed-rank test since our data has no normal distribution as tested with a Kolmogorow–Smirnow test. Results with *p* < 0.05 were considered statistically significant. A paired test was used since different methods were compared on the same patients. All statistical analyses were performed with python 3.8.

## 3. Results 

### 3.1. Study Cohort

The PACS crawler query for patients diagnosed with ICH by CT between January 2007–July 2021 yielded a total of 7733 subjects. Of these, 312 subjects did not have consent for the use of patient-related data for research purposes. Of the remaining 7421 subjects, 6121 had no or only an incomplete admission records with information on the symptom onset and thus no identifiable gold standard. Of the remaining 1300 datasets, 644 subjects fulfilled the final inclusion criteria (blood volume > 15 mm^3^ and onset > 1 h and <48 h) and thus were used for the training model. The study workflow is summarized in Figure 1. 

### 3.2. Onset Estimation of Classifiers and Human Raters

Both the AI-based classifiers and the radiologists had poor discrimination of the known onsets, with a mean absolute error (MAE) of 9.77 h (95% CI (confidence interval) = 8.52–11.03 h) for the convolutional neural network (CNN), 9.96 h (8.68–11.32 h) for the radiomics model, 13.38 h (11.21–15.74 h) for human rater 1 and 11.21 h (9.51–12.90 h) for rater 2, respectively (see Table 1).

Rater 1 and rater 2 were both significantly more inferior than the mean of the known onsets (*p* = 0.006 and *p* = 0.045, respectively). The results of the CNN and Radiomics model were both not significantly different to the mean of the known onsets (*p* = 0.705 and *p* = 0.423). The intraclass correlation between rater 1 and rater 2 was poor (intraclass correlation coefficient, ICC = 0.251).

## 4. Discussion

Our study shows that both human raters and machine learning algorithms only have poor discriminatory power to estimate the onset of patients with intracerebral hemorrhage based on CT imaging features. 

This may be partially explained by the nature and course of the disease. It is known that acute ICH may show mixed densities on CT, representing blood of different ages [20,21,22,30,31]. Thus, onset estimation based on density in CT may be misleading. Another feature, perihematomal edema, is known to progress over time but also depends on other factors such as anticoagulation status [32]. Further, several other factors such as patient age [33], anticoagulation medication [34], and blood pressure [35] are also known factors that impact imaging appearance of ICH beyond time. Regarding the manual ratings available studies have shown promising interrater agreements for ICH shape and density features [36,37]. However, so far, no published studies have found a rating based or machine learning algorithm that could reliably determine the onset of ICH patients with unknown symptom onset. 

Even though currently limited, therapeutic and triage decisions are based on factors such as the likelihood of rebleeding, brain edema and herniation which are associated with the time since onset of the bleeding [12]. Possible treatment strategies include blood pressure reduction [13,14], neurosurgical evacuation [16], and application of tranexamic acid [17,38], which may reduce the risk of hematoma expansion associated with neurological deterioration leading to poor outcomes and death [12,20]. These current approaches target hematoma expansion which is more likely to occur in the earlier time window [36]. In this context, prediction of the onset in patients with ICH and unknown onset may have had a great clinical relevance; however, the mean error of almost 10 h is probably too imprecise to be used for clinical decision making. Instead, other features that are likely to identify the risk of hematoma expansion will probably be used as an alternative. Also, for the prediction of hematoma growth and functional outcomes, machine learning algorithms have shown promising accuracy [23], most likely because several other features that can be extracted from imaging determine outcomes more than time since onset.

**Figure 2:**
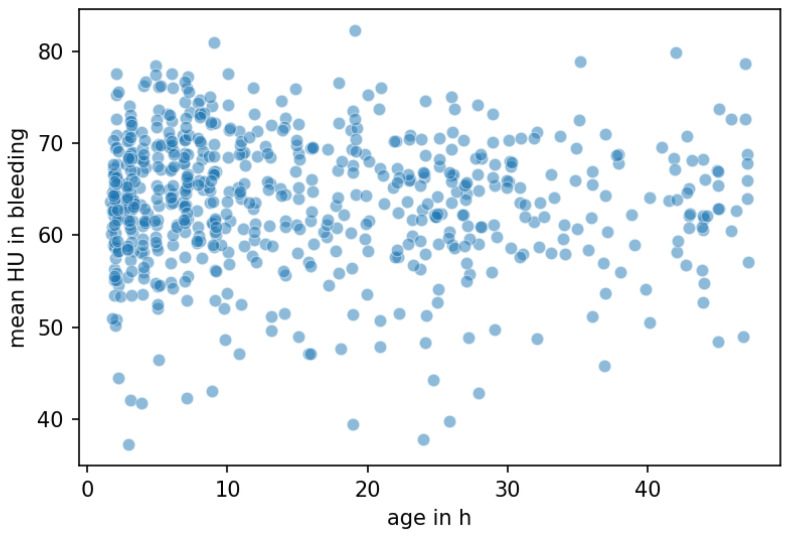
Mean density of intracerebral hemorrhage on admission imaging and association with symptom onset. HU = Hounsfield Units, age in h = time since onset in hours.

Our study has several limitations. First, the underlying ground truth (gold standard) of the timepoint of onset—on which the manual ratings and machine learning classifier are based—may be imprecise in some cases. Basically, the quality of the ground truth is a very important parameter for the performance and accuracy of an AI-based model. In other words, the AI model is only as good as the quality of the data on which it is based. Since the exact onset of ICH in our cohort could only be determined very imprecisely in part and depended on many factors (medical history, report by emergency physician, unclear clinical symptoms), the ground truth on which our AI-based model is based is also relatively imprecise. This was also supported by the fact that, considering the bleeding age of our total cohort, no decrease in density values (Hounsfield Units) could be reliably delineated over time (Figure 2). However, improving ground truth in general is problematic, as the exact onset of an ICH can never be determined with absolute certainty and will always be relatively inaccurate due to factors mentioned above.

In general, the acuity of clinical symptoms may be comparable to ischemic strokes, where reported symptom onsets are used for several studies that validated imaging-based approaches for onset estimation [39,40,41,42,43]. Second, it may be argued that a larger number of patients for training and validation of the classifier may have yielded more accurate results. Basically, the larger the dataset on which the algorithm is trained, the better AI-based methods perform. In other words, the data size of the cohort is a crucial factor. Some studies have shown that when the data size is very large, AI-based algorithms perform equally well or even better than the expert, for instance human rater [44]. In our training data set, only 644 subjects were included for the ICB onset estimation. It would therefore be interesting to see whether the results could be significantly changed or improved by including a significantly larger number of subjects. However, the current study already uses a comparably large dataset, and the results were still far from being clinically useful. Third, we must note that an average density (Hounsfeild Units) decrease of acute ICH would be expected over the time [45,46]. However, this is a crude measurement when used for shorter timespans of a few hours. Moreover, ICH often consists of blood of mixed densities which may bias the results even more [36]. Based on these factors and the above-mentioned results, it can be assumed that the information content of the CT scans in which ICH could be diagnosed does probably not contain or cover the information we are looking for (ICH onset). In our example, the human raters were also unable to read the bleeding age (onset time) from the CT dataset more accurately than approximately ± 10 h. For this reason, this information probably does not exist sufficiently in the image. Conversely, if the human rater already cannot confidently read out the information, the AI-based approach probably cannot either. In this context, only the analysis of extremely large and perfectly annotated datasets would be promising (see discussion above). Fourth, we did not use multimodal data for our AI-based approach, but only CT-based data. However, for ICH onset prediction, other data such as perfusion indicators [47], hemodynamic metrics [48], morphology and anatomical differences of intracranial arterial vessels especially with regard to microvascular collateral circulation [49,50] or CT-derived secondary hemodynamic parameters, and laboratory test results could be used and even combined. Such a multimodal AI-based approach could again significantly improve ICH onset prediction and should be considered in future studies. 

## 5. Conclusions

In our study, the discriminatory power of AI-based classifiers and human readers for onset estimation of patients with ICH was poor. This indicates that accurate AI-based onset estimation of patients with ICH based only on CT data may be unlikely to change clinical decision making in the near future. Perhaps multimodal AI-based approaches could improve ICH onset prediction and should be considered in future studies. 

## Figures and Tables

**Table 1:** Results of human raters and machine learning classifiers for onset estimation of patients with intracerebral hemorrhage.

	Mean Absolute Error (MAE) in h	95% Confidence Interval (CI)
Rater 1	13.38	11.21, 15.74
Rater 2	11.21	9.51, 12.90
CNN	9.77	8.52, 11.03
Radiomics	9.96	8.68, 11.32
Mean of known onset in entire cohort	9.81	8.62, 11.06

MAE in hours and confidence interval (95%) for human raters and AI-based models (CNN and Radiomics)

## Data Availability

The data presented in this study are available on request from the corresponding author.

## Abbreviations

AI	Artificial intelligence
CI	Confidence interval
CT	Computed tomography
GCS	Glasgow coma scale
ICH	Intracerebral hemorrhage
ICC	Intraclass correlation coefficient
INR	International normalized ratio
IVH	Intraventricular hemorrhage
mRS	Modified ranking scale
MAE	Mean absolute error
MRI	Magnetic resonance imaging
OAC	Oral anticoagulants
PACS	Picture Archiving and Communication System
HU	Hounsfield Unit
RIS	Radiological Information System
